# Single Step, Electrochemical Preparation of Copper-Based Positive Electrode for Lithium Primary Cells

**DOI:** 10.3390/ma11112126

**Published:** 2018-10-29

**Authors:** Maciej Ratynski, Bartosz Hamankiewicz, Michal Krajewski, Maciej Boczar, Dominika Ziolkowska, Andrzej Czerwinski

**Affiliations:** 1 Faculty of Chemistry, University of Warsaw, Pasteura 1, 02-093 Warsaw, Poland; mratynski@chem.uw.edu.pl (M.R.); mkrajewski@chem.uw.edu.pl (M.K.); mboczar@chem.uw.edu.pl (M.B.); daziolkowska.edu@gmail.com (D.Z.); 2 Biological and Chemical Research Centre, University of Warsaw, Zwirki i Wigury 101, 02-089 Warsaw, Poland

**Keywords:** lithium primary batteries, positive electrode, copper electrode

## Abstract

Lithium primary cells are commonly used in applications where high energy density and low self-discharge are the most important factors. This include small coin cells for electronics, power backup batteries for complementary metal-oxide-semiconductor memory or as a long-term emergency power source. In our study we present a fast, electrochemical method of the positive electrode preparation for lithium primary cells. The influence of the current density and oxygen presence in a solution on the preparation of the electrode and thus its electrochemical behavior is examined. Electrode compositions were characterized by X-ray photoelectron spectroscopy (XPS). The prepared electrodes may be used in Li cells as competition to Zn-MnO_2_ primary batteries.

## 1. Introduction

Lithium primary cell chemistry had begun in 1913, when G.N. Lewis presented a very accurate measurement of the lithium electrode potential [1]. In the early 1970s, the first commercial lithium battery was introduced to the market. Those cells present a great energy density and shelf life due to lithium metal use as an anode material. Metallic lithium has the standard potential close to −3.04 V vs. SHE (standard hydrogen electrode) [2] and theoretical specific capacity 3.86 Ah·g^−1^ [3]. A long shelf life of the lithium batteries is possible due to a stable, protective SEI (solid electrolyte interphase) layer at the metal surface. This layer made from the decomposed organic electrolyte protects the metallic anode from further unwanted reactions. The main drawback of the SEI layer is a limitation of the maximum current density due to its resistance and so-called “potential delay” at the beginning of the cell discharging.

Up to now many cathode materials were tested and commercialized. Liquid cathodes such as SOCl_2_, SO_2_Cl_2_, POCl_3_ and solid cathodes—CuO, CF_x_, FeS, AgCrO_4_, MnO_2_, V_2_O_5_, iodine-polyvinylpyridine are the most important examples [3,4]. However, only a few of them are currently produced at the large scale. Lithium-Iodine-Polyvinylpyridine battery is the main product for cardiac pacemakers. Li-Fluorocarbons and Li-MnO_2_ are main products for a “regular consumer” use. About 80% of all lithium primary cells in the market uses the manganese oxide cathode [5]. These batteries present a practical energy density per total cell mass of up to 300 Wh·kg^−1^ [5]. This is more than two times greater value than the popular primary alkaline Zn-MnO_2_ or Leclanche cells (up to 150 Wh·kg^−1^, 50 Wh·kg^−1^ per total cell mass respectively) [3].

Fluorocarbon (CF_x_), where x can vary from 0.4 to 1, is a partially fluorinated material made from coke (commercial), graphite, nanotubes etc. Lithium fluoride and carbon are discharging products, and thus this reaction is irreversible. Typically these batteries have a high energy density, but can produce a very low power because carbon fluorination greatly decreases their electrical conductivity. The Faradaic yield of this battery is also low (<75%), due to a presence of the CF_2_ and CF_3_ inactive groups [6]. To solve those problems MW-fluorinated nanotubes [7] or partially exfoliated, fluorinated graphite [8] are used. In those high energy compounds (>2200 Wh·kg^−1^), a non-fluorinated core provides an electric path to reaction centers. Similarly, the low electric conductivity is the crucial problem for MnO_2_ cathodes. A conductive carbon additive in cathode material is necessary for a higher current density output [9]. The MnO_2_-coated CNT (carbon nanotubes) structures were also successfully incorporated to increase a possible discharge current [10]. In most cases, both materials exhibit a low conductivity and maximum discharge rate below C/10 [5,8].

Today CuO electrodes are mainly researched in terms of the Li-ion secondary cell anode material. However, this approach requires a preparation of copper and carbon nanomaterials [11,12,13], which is often a slow and rather expensive process. In many situations the lab scale processes of the nanomaterial synthesis cannot be easily transferred to a commercial scale. Primary copper oxide cathodes are known for over 30 years [14] and exhibit an excellent capacity (2 times greater than Li-CF_x_ or Li-MnO_2_ systems), shelf life (less than 9% capacity loss per year at 90 °C) and very good operation safety, whereas a current density is still an unsolved issue. Here we present a novel, fast, electrochemical method of the Cu-based electrode preparation. Optimal formation conditions provide the ability of the fast discharge without a need for conductive additives. CuO has the theoretical capacity and energy density 670 Ah·kg^−1^ and 1280 Wh·kg^−1^, respectively. Furthermore, the operating voltage of the Li-CuO cell is close to 1.5 V [3].

## 2. Materials and Methods

The positive electrodes were prepared by an electrochemical, galvanostatic oxidation of the copper foil in 2.5 M sodium hydroxide (NaOH) solution. The foil oxidation was prepared at the current density of 1.5 mA·cm^−2^ (sample B, C) and 3.0 mA·cm^−2^ (sample A). The total charge density passed through the foil was set to 4.5 C·cm^−2^. A constant argon flow through the solution was set 30 min. before sample C preparation and lasted during a whole sample C processing. Then the foil was removed and washed with deionized water three times to remove NaOH solution, and dried at 80 °C and manually cut.

Electrochemical tests were performed at Solatron 1287 (cyclic voltammetry) and Atlas 0961 (galvanostatic discharge). The positive electrode was cut from the oxidized foil and used without further preparations. Lithium metal was used as the negative and reference electrodes. 1 M LiPF_6_ in EC/DMC 1:1 solution was used as the electrolyte. Cells were set up in 3 electrode Swagelok^®^ case.

Carl Zeiss field emission scanning electron microscope (FE-SEM, Merlin model, Carl Zeiss Oberkochen, Germany) was used to obtain microscopic images. Images were recorded by in-lens secondary electron (SE) detector. Electron high tension acceleration voltage was set at 3 kV.

The powder X-ray diffraction (XRD) analysis was performed in order to identify the crystal structure, determine the crystallite sizes, and measure unit cell parameters. Bruker Discovery D8 XRD system (Bruker, Billerica, MA, USA) with a non-monochromatic Cu Kα radiation (λ = 0.15406 nm) was used for this study. The XRD patterns were collected in the 2-Theta coupled mode with the step size of 0.0244°. The average crystallite size was determined based on the Scherrer formula and the unit cell parameters were obtained from the Rietveld refinement analysis performed using DIFFRAC.EVA (Bruker) software (software version 2.0, Bruker, Billerica, MA, USA).

X-ray photoelectron spectroscopy (XPS) experiments have been performed using Kratos Axis Supra instrument (Kratos Analytical Ltd., Manchester, UK) with Al Kα monochromatic beam (1486.7 eV) as an X-ray source. The X-ray takeoff angle was set to a standard 45°. The pass energy was set to 160 eV and 20 eV for low and high resolution spectra acquisition providing an energy resolution of ~7.5 and ~0.8 eV respectively. The data treatment was performed within the CasaXPS software (software version 2.3.19, Casa Software Ltd., Teignmouth, UK). All spectra were calibrated by using C 1s adventitious carbon as a reference binding energy (284.8 eV).

## 3. Results and Discussion

### 3.1. XRD Measurements

**Figure 1:**
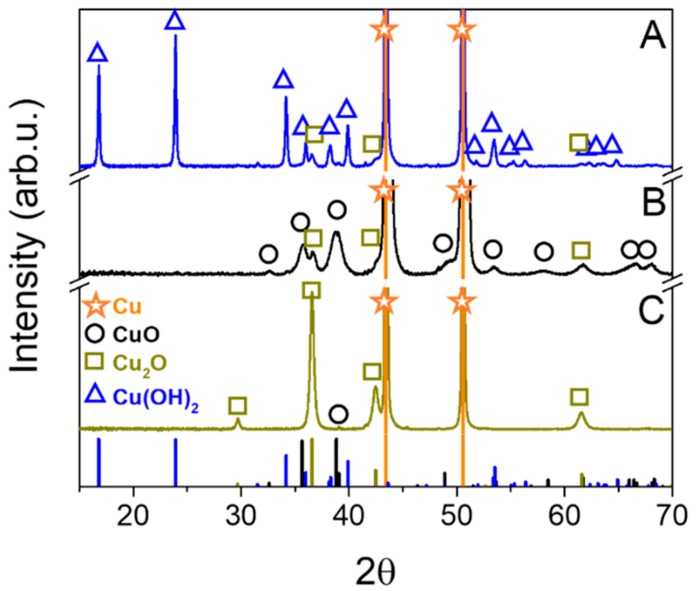
XRD patterns of electrodes A, B and C.

The crystal structure of the materials was examined by a X-ray powder diffraction method. This analysis showed that each electrode sample crystallized in different composition on Cu support at the given current and atmosphere (Figure 1). The sample A layer is composed mostly of Cu(II) hydroxide (Cu(OH)_2_) orthorhombic crystal structure (Cmcm (63) space group). The average crystal size of this phase estimated from the Scherrer equation was about 52 nm. The refined lattice parameters were: a = 2.94 Å, b = 10.55 Å, and c = 5.24 Å. The small amount (9%) of Cu(I) oxide (Cu_2_O) phase was also detected. Samples B and C showed completely different diffraction patterns. The main phase of the sample C was Cu_2_O crystallized in a cubic structure (Pn-3m (224) space group) with the lattice parameter a = 4.25 Å. Its estimated crystal size was about 23 nm. This suggests that the constant argon flow through the solution during the oxidation process created optimal conditions for Cu metal oxidation into Cu(I). This sample had also barely detectible traces of Cu(II) oxide (CuO) crystals (~1%). The last sample B crystallized in two phases: Mainly CuO structure with an addition of 13% Cu_2_O. The cell parameters of CuO monoclinic crystal structure (C2/c (15) space group) were following: a = 4.70 Å, b = 3.42 Å, c = 5.13 Å, α = γ = 90°, and β = 99.54°. The average crystallite sizes of CuO and Cu_2_O phases were: ~8 nm and ~17 nm, respectively. This suggests that a lower current density during the foil oxidation caused a lower crystallization degree (a slower nucleation and growth of crystals). There is an expected preferential crystal growth observed through a selective increase of the (021) plane intensity. The samples compositions and crystal sizes are summarized in Table 1.

### 3.2. XPS Measurements

**Figure 2:**
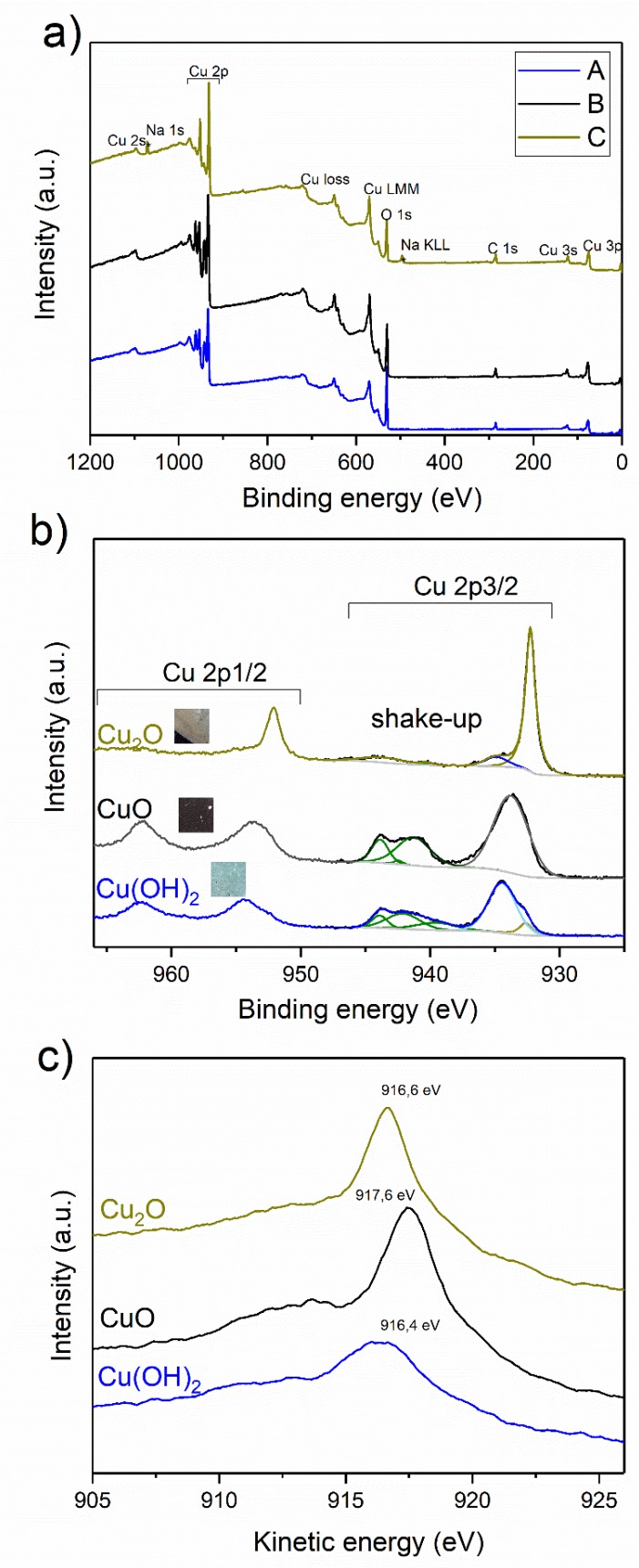
XPS analysis of electrodes A, B and C: (**a**) survey scan; (**b**) Cu 2p high resolution spectra; and (**c**) X-ray induced Auger Cu LMM spectra.

The optical examination of the samples after the preparation shows three different colors of the formed layer (blue, black and dark yellow). Colors depended on formation conditions: Current density and oxygen presence in a solution. These samples were labeled as A, B, C, respectively. Figure 2a. represents the XPS wide scan spectrum of three electrodes (A, B and C). The spectra comprise several main peaks which correspond to copper, oxygen and adventitious carbon. The spectrum of the sample C also reveals the presence of sodium in the material: The Na 1s peak at binding energy (BE) 1071 eV and Na KLL Auger line at 496 eV, which is the result of material contamination during the electrode preparation. The relative surface concentrations of main species present in three samples are listed in Table 2.

It can be observed that copper and oxygen concentrations in all materials were in the range of 20–30% and 40–50% respectively. The O/Cu ratio indicates that the most of surface-oxidized copper is present in the A sample. Figure 2b depicts the high-resolution spectra of Cu 2p region which basically comprise of two components—Cu 2p3/2 and Cu 2p1/2. Highly-intense shake-up satellites can be also observed a at A and B samples spectra. Shake-up peaks may occur when the outgoing photoelectron simultaneously interacts with a valence electron and excites it to a higher-energy level. The kinetic energy of the shaken-up core electron is then slightly reduced giving a satellite structure a few eV below (higher on the calculated binding energy scale) the core level position [15]. This phenomenon can be observed when Cu(II) is excited by X-rays and does not occur in the case of metallic copper or Cu(I). On this basis one can conclude that samples B and C comprises mostly of Cu(II) species. The shake-up satellite is also observed for electrode A, but it is much less intense, as previously reported [15]. Table 3 presents the data of Cu 2p3/2 spectra curve fitting for all three electrodes. The binding energies of the main component are 934.4, 933.8, and 932.3 eV for A, B, and C samples, respectively. Two materials (A and C) reveal a doublet at the spectra, which indicates its complex nature (12% and 21% of minor signals, respectively). The binding energy of minor peaks are 932.6 and 934.9 eV for A and C samples. According to NIST XPS Database [16] a very small chemical shift of Cu 2p3/2 peak for different copper species unable to assign correct chemistry only based on Cu 2p3/2 binding energy. This can be overcome by determination of Auger parameter, which is a calculated value from both photoelectron and Auger peak positions (Cu 2p3/2 and Cu LMM in case of copper). This parameter is particularly useful for a chemical state analysis and can be used without an interference of the surface charging [17]. Figure 2c represents the X-ray induced Auger spectra of the Cu LMM line. The kinetic energy of these peaks are 961.4, 917.6 and 916.6 eV for A, B, and C samples, respectively. Table 4 lists the calculated Auger parameters (the sum of binding Cu 2p3/2 and kinetic Cu LMM energies) of A, B and C electrodes. The sample A (blue electrode) composes mostly (88%) of copper hydroxide (minor Cu(I) oxide), the sample B (black electrode) is a pure Cu(II) oxide and the sample C is composed of Cu(I) oxide (79%) with 21% of Cu(II) oxide. The difference between atomic composition determined by XRD and XPS may result from their various detection depths. As XPS is surface-sensitive technique, one can observe that the outer layer of all analyzed materials is richer in oxidized species due to its air exposure before experiments.

XPS results confirmed that a higher oxidation current density resulted mainly in a Cu(II) hydroxide formation, while a lower current created Cu(II) oxide. The oxygen removal from the solution by argon bubbling at the low oxidation current led to Cu(I)oxide as the main product.

### 3.3. Electrochemical Measurements

#### 3.3.1. Cyclic Voltammetry

**Figure 3:**
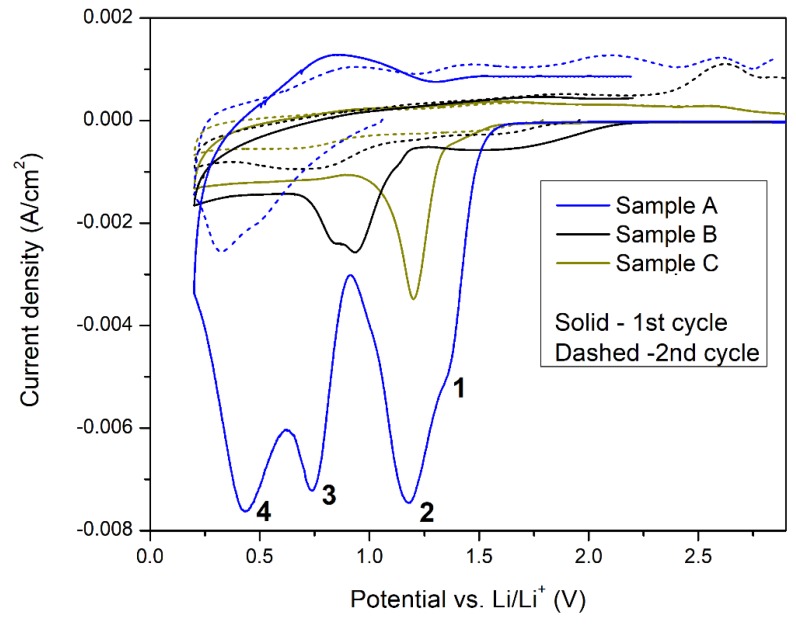
Voltammetry for scan no. 1 and no. 2 for sample A, B, C.

**Figure 4:**
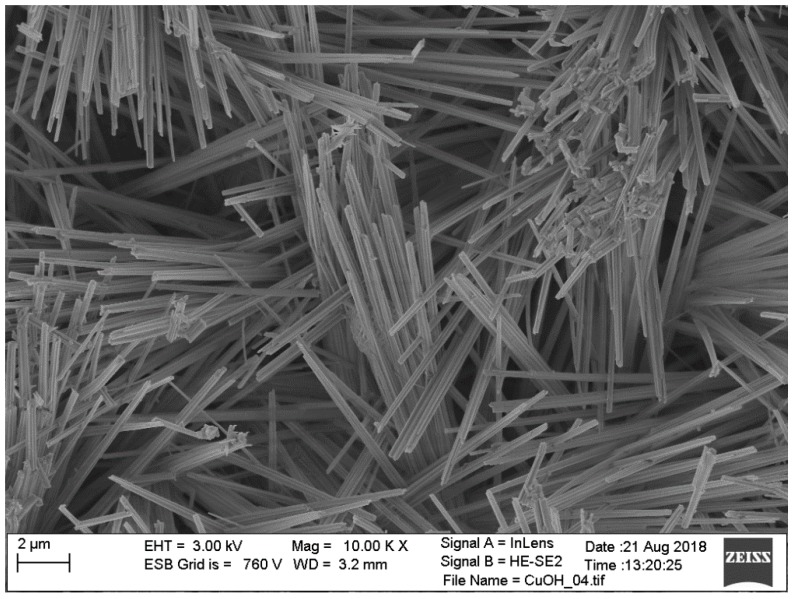
SEM image of sample A.

Cyclic voltammetry measurements were performed at 1 mV·s^−1^ scan speed. The first (solid line) and second (dashed line) voltammetry cycles of freshly combined cells (sample A, B and C) are presented in Figure 3. A small and diffuse oxidation peak is observable only for sample A, but is completely missing in samples B and C, suggesting that the ongoing lithiation reaction is almost irreversible for all examined materials. The second voltammetry scan only confirmed irreversibility of the lithiation in those samples. There was no significant oxidation peak visible up to potential equal to 2.8 V vs. Li/Li^+^ for sample C and only small oxidation peaks were observed for samples A and B. A peak current density and total charge were significantly lower compared to reduction peaks indicating a reaction predominant irreversibility. This differs from observation for Cu_2_O nanorods [18], CuO nanostructures [19,20,21] or Cu(OH)_2_ nanoflowers [22], where significant oxidation peak was present at 2.2–2.7 V vs. Li/Li^+^. The lithiation reversibility in metal oxides/hydroxides is strongly affected by their morphology. Nanostructures present a much better reversibility than macro-deposits. The effect is even stronger when nanostructures are dispersed in conductive carbon/graphene matrix [23,24,25]. MnO_2_ reversible lithiation was successfully done (despite large oxide volume changes during lithiation), when a thin metal oxide layer is deposited on the CNT surface [10]. No supporting/conductive material was added in this study and thus the reversibility was not observed. The morphology of the sample A (which presents the highest reduction potential and peak current density at CV scan) was examined by SEM imaging. A representative image of the sample A is presented in Figure 4. The deposited Cu(OH)_2_ formed long (over 10 µm), but very thin (below 200 nm) sharp, needle-like structures. A very poor lithiation reversibility may be the result of such sophisticated material morphology. After the lithiation, the needle-like structure should collapse due to different crystal structure of Cu(OH)_2_ and formed LiOH. In such case the freshly formed lithium hydroxide will lose the electric contact with current collector that is covered by a resistive SEI layer. The intense formation of the SEI layer at a potential below 0.5 V vs Li/Li^+^ is confirmed by large peak visible at CV scan (peak no. 4 in Figure 3). The combination of these two factors: Structure collapsing and electric pathways blocking by SEI layer, results in kinetic limitations of material oxidation, and thus reduces the reaction reversibility. A much better reversibility obtained for similar compounds by other authors was achieved by blending the copper oxide/hydroxide with conductive carbon and polymeric binder material. A dense and compact arrangement of the material particles prepared in this process increases the electric conductivity of the electrode during cycling and improves reaction reversibility. However, this approach greatly limits the electrolyte access to the active material which is a key factor for a high discharge current density.

Interestingly, CV results (Figure 3) revealed multiple reduction peaks from samples A and B and single reduction peak from the sample C during the first voltammetry scan. At the second voltammetry scan (dashed lines in Figure 3) a significant decrease of the reduction peak current density was observed for all samples. The cathodic scan for the sample C shows one reduction peak which is correlated to a reduction of Cu_2_O to Cu and a formation of Li_2_O. Similarly, the sample B reduction peak originates from reduction of CuO to Cu. A small amount of Cu_2_O present in this sample gives two peaks in this scan, at approx. 0.9 V and 0.8 V. Two peaks at 0.99 V and 0.74 V for CuO nanostructure reduction were previously obtained by L. Shi et al. [26]. A multi-step electrochemical reduction of CuO to Cu and Li_2_O was proposed in that report; the intermediate products of this process were Cu_1−x_^II^Cu_x_^I^O_1−x/2_ and Cu_2_O phases. A results revealed by sample “A” suggest a gradual multistep reduction of Cu(OH)_2_ to Cu_2_O through formation of LiOH and Cu_x_O_(2>x>1)_ intermediate compound [22]. Peak “1” at ~1.4 V can be attributed to small amounts of Cu(I) reduction (<12% of Cu_2_O found on the surface and 9% in the bulk by XPS and XRD, respectively). Peak “2” at 1.2 V originates from Cu(OH)_2_ to Cu_x_O_(2>x>1)_ reaction and further to metallic Cu at 0.75 V (peak “3”). Peak “4” at 0.4 V is related to SEI layer growth due to electrolyte carbonates decomposition. This peak is less visible for samples B and C probably due to a low real surface area of these electrodes. Peak potentials for individual reactions e.g., Cu(I) or Cu(II) reduction are different in sample A, B and C. This may originate from a different structure of oxides in each sample. L. Shi et al. [26] and C. Wang et al. [19] also observed the copper oxide structure dependence on reduction peak potential. The CuO reduction may change over 140 mV for different oxide nanostructures.

#### 3.3.2. Galvanostatic Discharge

The presence of the large peaks at 0.9–1.5 V suggests the applicability of the Cu(OH)_2_ nanomaterial as a primary lithium cell cathode; Li/Cu(OH)_2_ cell working voltage range is compatible with 1.5 V Zn/MnO_2_ primary cells or NiMH secondary cells widely used in almost every portable application. Thus sample A was used for galvanostatic discharge capacity measurements. The cell was discharged at 1 mA current (~0.6 C based on post-measured discharge time). The total electrode mass including current collector was used for a current density and capacity calculations. The capacity (*C*) was determined using following formula:

(1) $$\;C=\frac{I\times t}{M}\;$$

where *I* is an applied current, *t* is a discharge time, and *M* is a total mass of the electrode (current collector with active material).

**Figure 5:**
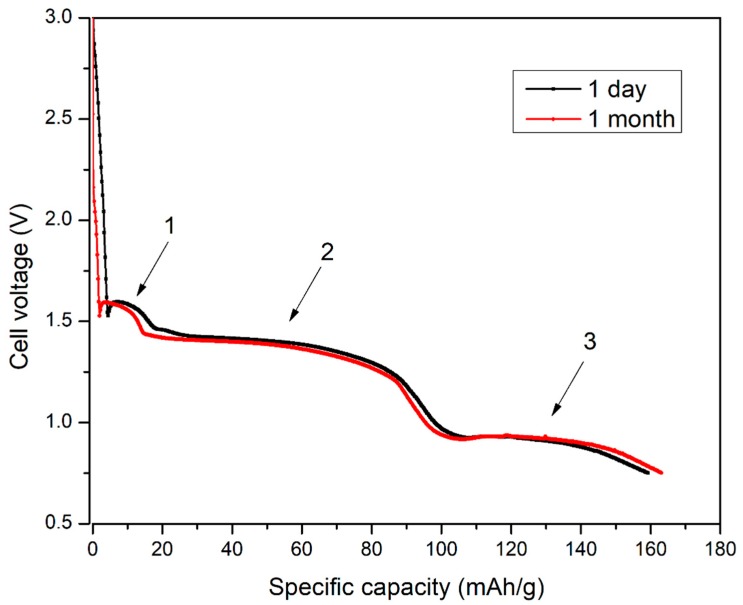
Cell voltage vs. Specific capacity at galvanostatic discharge test after 1 day and 1 month storage.

The discharging rate was high enough to maintain the current density for modern, high power devices. The discharging curve (Figure 5) revealed a presence of three plateaus correlated with the reaction steps observed in cyclic voltammetry measurement. A fully charged cell operated at voltage close to 1.6 V which is close to the modern zinc-carbon primary cell voltage. A steady voltage profile (close to 1.4 V) was achieved up to 90 mAh·g^−1^ followed by voltage drop to 0.95 V up to 150 mAh·g^−1^. SEI layer formation (observed in CV experiment as peak “4”—Figure 3) was not present at galvanostatic experiment because discharge end voltage was set to 0.75 V.

Plateaus at 1.6 V and 0.95 V could be used as an internal capacity indicator. Typically, a battery voltage decreases slowly and monotonically in a wide state of charge (SoC) range and then very rapidly at the low SoC region (<5%) giving very inaccurate information about the SoC until the battery is almost fully discharged. In our system, the user could easily check whether the battery is new (1.6 V) or used based on whether it indicates less than 60% (1.4–1.3 V) or over 70% (0.95 V). The capacity at the last plateau (over 30% of the overall cell capacity) gives the time for the new battery purchase. A similar concept was used in first Li-CuS batteries applied as a cardiac peacemaker power supply since 1976. CuS batteries have two plateaus close to 2.12 V and 1.75 V. The theoretical capacity of the CuS material is 560 Ah·kg^−1^ [3]. The Cu(OH)_2_ theoretical capacity is very similar and close to 550 Ah·kg^−1^. Practical energy densities of these cathodes in lithium primary cells are however hard to compare, because Li-CuS cells are mainly classical bobbin cells designed for a low power operation, while our cell is designed for a spirally wounded battery and can operate at the high power output.

One of the most important and common factor affecting a commercial use of the primary lithium cells is their self-discharge. To test this parameter, the cell was kept at ambient conditions for 31 days. This test revealed a very low energy loss during this period. Calculations showed 193.6 ± 0.6 Wh·kg^−1^ for freshly assembled and 192.2 ± 0.6 Wh·kg^−1^ for the 1 month old cell. A total energy density loss was assumed to be 0.7 ± 0.5% per month. The obtained practical energy density (per cell mass) is lower, when compared with other primary lithium systems such as lithium-fluorocarbons (230–300 Wh·kg^−1^), lithium-manganese oxide (155–230 Wh·kg^−1^ for spirally wound cell), or lithium-copperoxide (280 Wh·kg^−1^ at C/1000 current) [3]. The theoretical maximum capacities of these compounds are 860 Ah·kg^−1^, 310 Ah·kg^−1^ and 670 Ah·kg^−1^ [3]. The Cu(OH)_2_ theoretical capacity is close to 550 Ah·kg^−1^ which is closely comparable with these materials. The practical capacity (Figure 5) and energy density of our system is lower than these values, but our cell, in contrast to most of designs [3,5,8,27], is suitable for a high current density operation.

Our cell, as one of the few examples, were tested using high current (0.6 C), while many primary systems are designed to work at low currents. Typical CR-type primary batteries with MnO_2_ [5] or exfoliated fluorinated graphite [8] are not capable of discharging (and maintain reasonable capacity) at currents exceeding 0.05 C and 0.1 C respectively. Currents lower than C/100 are typically used.

In contrast with many researchers our calculations were based on a total electrode mass (including current collector). It is common for the thin, light, active material layer to present an excellent capacity per active material mass, although it often also presents a very low per total electrode mass. To emphasize commercial application capabilities, we present more realistic capacity calculations (per total electrode mass).

## 4. Conclusions

We successfully developed a fast (only 25 min long) and easy, single step electrochemical method for a primary lithium cell cathode manufacturing. Different process conditions resulted in various material chemistries, further studied by XRD and XPS measurements to evaluate their bulk and surface structures. The best obtained material was prepared using a high deposition current and a presence of oxygen. This procedure led to the formation of Cu(OH)_2_ layer on Cu support. The lithiation of this material proceeds in three reduction steps at a voltage range of 1.6–0.8 V. The working voltage implies that this system can replace primary cells in many applications such as a commercial Zn-MnO_2_ battery. The practical energy density of our system is 192 Wh·kg^−1^ (based on a total electrode mass including current collector). This result is similar compared to an alkaline zinc-manganese oxide system (~150 Wh·kg^−1^ for bobbin cell) and with Li-MnO_2_ spirally wound cell (155–230 Wh·kg^−1^); Among these systems only our cell is capable of providing a high current output. The shelf life of our cell is also comparable or better than other primary systems. Energy loss after 30 days was very low—less than 0.7 ± 0.5%. A practical discharge current density equal to 0.6 C enables use in high-power devices. Based on a short preparation time, low cost and good electrochemical parameters, this system has a high commercialization potential. 

The procedure for material preparation and electrode based on material presented in this article had been patented in Patent Office of The Republic of Poland. Patent application number: P.426331.

## 5. Patents

The procedure for material preparation and electrode based on material presented in this article had been patented in Patent Office of The Republic of Poland. Patent application number: P.426331.

## Figures and Tables

**Table 1:** Deposited layer phase compositions of samples A, B and C and their crystal sizes. (bdl = below detection limit).

Sample	Deposited Layer Phase Composition (%)			Crystal Size of Individual Compounds (Å)		
	Cu(OH)_2_	Cu_2_O	CuO	Cu(OH)_2_	Cu_2_O	CuO
A	91	9	-	522	233	-
B	-	13	87	-	167	81
C	-	99	1	-	232	bdl

**Table 2:** Atomic concentrations of detected elements in samples A, B and C.

Sample	Atomic Concentration (%)				O/Cu Ratio
	Cu	O	C	Na	
A	23.1	51.8	25.1	-	2.2
B	30.1	43.2	26.7	-	1.4
C	22.1	38.3	37.0	2.6	1.7

**Table 3:** Binding energies of Cu 2p3/2 spectra.

Sample	Binding Energy Cu 2p3/2 (eV)		% of Main Component
	Component 1	Component 2	
A	934.4	932.6	88
B	933.8	-	100
C	932.3	934.9	79

**Table 4:** Kinetic energies and Auger parameters of species detected in A, B and C electrodes.

Sample	Kinetic Energy (eV) Cu LMM	Auger Parameter (eV)		Assigned Chemistry	
		Component 1	Component 2	Component 1	Component 2
A	961.4	1850.8	1849.0	Cu(OH)_2_	Cu_2_O
B	917.6	1851.4	-	CuO	
C	916.6	1848.9	1851.5	Cu_2_O	CuO

## References

[1] Lewis G.N., Keyes F.G. (1913). The potential of the lithium electrode. J. Am. Chem. Soc..

[2] Huston R., Butler J.N. (1968). The Standard Potential of the Lithium Electrode in Aqueous Solutions. J. Phys. Chem..

[3] Reddy T.B., Linden D. (2011). Linden’s Handbook of Batteries.

[4] Garche J. (2009). Encyclopedia of Electrochemical Power Sources.

[5] Manane Y., Yazami R. (2017). Accurate state of charge assessment of lithium-manganese dioxide primary batteries. J. Power Sources.

[6] Hamwi A., Guérin K., Dubois M. (2005). Fluorinated Materials for Energy Conversion.

[7] Ahmad Y., Dubois M., Guerin K., Hamwia A., Flahaut E. (2017). High energy density of primary lithium batteries working with sub-fluorinated few walled carbon nanotubes cathode. J. Alloys Compd..

[8] Mar M., Ahmad Y., Guérin K., Dubois M., Batisse N. (2017). Fluorinated exfoliated graphite as cathode materials for enhanced performances in primary lithium battery. Electrochim. Acta.

[9] Frysz C.A., Shui X., Chung D.D.L. (1996). Carbon filaments and carbon black as a conductive additive to the manganese dioxide cathode of a lithium electrolytic cell. J. Power Sources.

[10] Reddy A.L.M., Shaijumon M.M., Gowda S.R., Ajayan P.M. (2009). Coaxial MnO_2_/Carbon nanotube array electrodes for high-performance lithium batteries. Nano Lett..

[11] Park S.-H., Lee W.-J. (2015). Hierarchically mesoporous CuO/carbon nanofiber coaxial shell-core nanowires for lithium ion batteries. Sci. Rep..

[12] Wang B., Wu X.-L., Shu C.-Y., Guo Y.-G., Wang C.-R. (2010). Synthesis of CuO/grapheme nanocomposite as a high-performance anode material for lithium-ion batteries. J. Mater. Chem..

[13] Zhang J., Wang B., Zhou J., Xia R., Chu Y., Huang J. (2017). Preparation of advanced CuO nanowires/functionalized graphene composite anode material for lithium ion batteries. Materials.

[14] Grimm M. (1986). Lithium copper oxide lithium copper oxyphosphate battery systems. IEEE Trans. Consum. Electr..

[15] Biesinger M.C., Lau L.W.M., Gerson A.R., Smart R.S.C. (2011). Resolving surface chemical states in XPS analysis of first row transition metals, oxides and hydroxides: Sc, Ti, V, Cu and Zn. Appl. Surf. Sci..

[16] NIST X-Ray Photoelectron Spectroscopy Database. https://srdata.nist.gov/xps/.

[17] Wagner C.D. (1972). Auger lines in x-ray photoelectron spectrometry. Anal. Chem..

[18] Chen W., Zhang W., Chen L., Zeng L., Wei M. (2017). Facile synthesis of Cu_2_O nanorod arrays on Cu foam as a self-supporting anode material for lithium ion batteries. J. Alloys Compd..

[19] Wang C., Higgins D., Wang F., Li D., Liu R., Xia G., Li N., Li Q., Xu H., Wu G. (2014). Controlled synthesis of micro/nanostructured CuO anodes for lithium-ion batteries. Nano Energy.

[20] Dang R., Jia X., Wang P., Zhang X., Wang D., Wang G. (2017). Hydrothermal synthesis of peony-like CuO micro/nanostructures for high-performance lithium-ion battery anodes. Chin. Chem. Lett..

[21] Liu X., Liu G., Wang L., Li Y., Ma Y., Ma J. (2016). Morphology- and facet-controlled synthesis of CuO micro/nanomaterials and analysis of their lithium ion storage properties. J. Power Sources.

[22] Pramanik A., Maiti S., Mahanty S. (2014). Metal hydroxides as a conversion electrode for lithium-ion batteries: A case study with a Cu(OH)_2_ nanoflower array. J. Mater. Chem. A.

[23] Peng H.-J., Hao G.-X., Chu Z.-H., He C.-L., Lin X.-M., Cai Y.-P. (2017). Mesoporous spindle-like hollow CuO/C fabricated from a Cu-based metal-organic framework as anodes for high-performance lithium storage. J. Alloys Compd..

[24] Lu L.Q., Wang Y. (2012). Facile synthesis of graphene-supported shuttle- and urchin-like CuO for high and fast Li-ion storage. Electrochem. Commun..

[25] Xia H., Zhang J., Chen Z., Xu Q. (2018). 1D Cu(OH)_2_ nanorod/2D SnO_2_ nanosheets core/shell structured array: Covering with graphene layer leads to excellent performances on lithium-ion battery. Appl. Sur. Sci..

[26] Shi L., Fan C., Sun C., Ren Z., Fu X., Qian G., Wang Z. (2015). Synthesis of different CuO nanostructures from Cu(OH)_2_ nanorods through changing drying medium for lithium-ion battery anodes. RSC Adv..

[27] Chen W., Zhang H., Ma Z., Li S., Li Z. (2018). Binder free Cu(OH)_2_/CuO electrodes fabricated directly on copper foils by facile large-scale production method. J. Alloys Compd..

